# Leveraging the Black Girls Run Web-Based Community as a Supportive Community for Physical Activity Engagement: Mixed Methods Study

**DOI:** 10.2196/43825

**Published:** 2023-09-07

**Authors:** Jolaade Kalinowski, Christie Idiong, Loneke Blackman-Carr, Kristen Cooksey Stowers, Shardé Davis, Cindy Pan, Alisha Chhabra, Lisa Eaton, Kim M Gans, Jay Ell Alexander, Sherry Pagoto

**Affiliations:** 1 Department of Human Development and Family Sciences The University of Connecticut Storrs, CT United States; 2 Department of Allied Health Sciences The University of Connecticut Storrs, CT United States; 3 Department of Nutritional Sciences The University of Connecticut Storrs, CT United States; 4 Department of Allied Health The University of Connecticut Storrs, CT United States; 5 Department of Communication The University of Connecticut Storrs, CT United States; 6 Black Girls Run Richmond, VA United States

**Keywords:** physical activity, social media, women’s health, African American women, mHealth, mobile health, Facebook, African American, exercise, web-based community, web-based communities, content analysis

## Abstract

**Background:**

About 59%-73% of Black women do not meet the recommended targets for physical activity (PA). PA is a key modifiable lifestyle factor that can help mitigate risk for chronic diseases such as obesity, diabetes, and hypertension that disproportionately affect Black women. Web-based communities focused on PA have been emerging in recent years as web-based gathering spaces to provide support for PA in specific populations. One example is Black Girls Run (BGR), which is devoted to promoting PA in Black women.

**Objective:**

The purpose of this study was to describe the content shared on the BGR public Facebook page to provide insight into how web-based communities engage Black women in PA and inform the development of web-based PA interventions for Black women.

**Methods:**

Using Facebook Crowdtangle, we collected posts (n=397) and associated engagement data from the BGR public Facebook page for the 6-month period between June 1, 2021, and December 31, 2021. We pooled data in Dedoose to analyze the qualitative data and conducted a content analysis of qualitative data. We quantified types of posts, post engagement, and compared post types on engagement: “like,” “love,” “haha,” “wow,” “care,” “sad,” “angry,” “comments,” and “shares.”

**Results:**

The content analysis revealed 8 categories of posts: shout-outs to members for achievements (n=122, 31%), goals or motivational (n=65, 16%), announcements (n=63, 16%), sponsored or ads (n=54, 14%), health related (n=47, 11%), the lived Black experience (n=23, 6%), self-care (n=15, 4%), and holidays or greetings (n=8, 2%). The 397 posts attracted a total of 55,354 engagements (reactions, comments, and shares). Associations between the number of engagement and post categories were analyzed using generalized linear models. Shout-out posts (n=22,268) elicited the highest average of total user engagement of 181.7 (SD 116.7), followed by goals or motivational posts (n=11,490) with an average total engagement of 160.1 (SD 125.2) and announcements (n=7962) having an average total engagement of 129.9 (SD 170.7). Significant statistical differences were found among the total engagement of posts (χ_7_^2^=80.99, *P*<.001), “like” (χ_7_^2^=119.37, *P*<.001), “love” (χ_7_^2^=63.995, *P*<.001), “wow” (χ_7_^2^=23.73, *P*<.001), “care” (χ_7_^2^=35.06, *P*<.001), “comments” (χ_7_^2^=80.55, *P*<.001), and “shares” (χ_7_^2^=71.28, *P*<.001).

**Conclusions:**

The majority of content on the BGR Facebook page (n=250, 63%) was focused on celebrating member achievements, motivating members to get active, and announcing and promoting active events. These types of posts attracted 75% of total post engagement. BGR appears to be a rich web-based community that offers social support for PA as well as culturally relevant health and social justice content. Web-based communities may be uniquely positioned to engage minoritized populations in health behavior. Further research should explore how and if web-based communities such as BGR can be interwoven into health interventions and health promotion.

## Introduction

Recent evidence suggests that 59%-73% of Black women are not meeting the recommended targets for physical activity (PA) [1]. Sedentary lifestyle is associated with obesity [2], diabetes [3], hypertension [4], and poor sleep quality [5], all of which disproportionately affect Black women. PA has been shown to reduce the risk for these conditions; yet uptake is particularly low among Black women [6-8].

Black women face a myriad of barriers to PA, such as low social support [9], low access to green spaces [10], and safe places to exercise [11]. Cultural norms and body politics play an integral role in Black women’s body image, self or external reflection, and can pose as barriers to PA [12]. For some, PA is associated with a desire for weight loss and can be a motivating factor to engage in PA. But for others, lower body satisfaction and body image may impede motivation to engage in PA [13]. Other research suggests that acceptance of larger body size may also dissuade engagement in PA [14].

It is important to consider the historical context for the complex nature of body image, beauty standards, and stereotypes about Black women [15]. The exploitation of Saartje Baartman, a Black woman with steatopygia, which is a condition that is characterized by high-fat proportions in thighs and buttocks [16], had a profound impact on the expectations of Black women and their bodies [17,18]. The effects of her exploitation had a lasting legacy on how contemporary Black women view beauty standards and the promotion of the curvy body type [19]. Similarly, the “mammy” stereotype of Black women, which portrays Black women as large and strong, with a purpose to serve others, has also had profound implications on wellness, body image, and expectations of Black women in and outside of the Black community [20,21]. Emerging qualitative research also underscores the influence and legacy of these cultural stereotypes and their implications on health, body ideals, and preferences and expectations regarding weight [22], which also has implications for attitudes toward PA [12,23].

Despite these nuanced historical factors, web-based Black communities are emerging as potentially safe spaces to congregate and support one another around physical and mental health [24,25]. Emerging evidence suggests that social support and social networks may be especially critical in engaging Black women in PA [26-28]. One example is Black Girls Run (BGR), a web-based recreational running community for Black women. BGR provides an opportunity for Black women to run with others that “look like you” in a predominantly White space while simultaneously challenging dominant narratives about Black women’s bodies, PA, and health [29]. Established in 2009, the mission of BGR is to motivate Black women to prioritize fitness and healthy lifestyle through running. Across the nation, BGR has 75 local chapters, many of which have their own Facebook groups and pages [30]. BGR’s national Facebook page has attracted 231,785 followers and 215,089 likes. The national BGR Facebook page is public such that any Facebook user can view and engage with its content, which could result in their content appearing in that users’ newsfeed.

The emergence and growth of health-focused web-based communities such as BGR are exemplary of emerging evidence that social media may be an effective tool for engaging Black women around health topics [31]. One study of smartphone use and social media engagement found that Facebook usage among Black women was as high as 80%, depending on age [32]. Yet, very little research has been done to examine large, highly engaged social media communities such as BGR, including how they engage members, the characteristics of posts, and what type of content attracts the most engagement from the community. As such, we performed a content analysis of 6 months of Facebook posts on the BGR national Facebook page. Then, we compared post types on engagement to determine the type of content that elicited the most engagement. This study may provide insight into how web-based communities engage Black women in PA and may inform the development of web-based PA interventions for Black women.

## Methods

### Data Collection

Methods were largely derived from previous investigations using social media data [33,34]. Similar to the previous studies, we used CrowdTangle Search, a service owned by Meta that allows researchers to extract publicly available data on Facebook [35,36]. On January 11, 2022, we extracted all Facebook posts in the BGR Facebook page between the 6-month period of June 1, 2021, through December 31, 2021. We also collected data on post engagement, which included reactions (eg, likes), comments, and shares. We exported the posts and engagement data to an Excel (Microsoft Corp) spreadsheet and then Dedoose for content analysis.

### Ethical Considerations

We followed similar methodology and processes as previous social media research and content analysis studies [33,37-39]. This study was deemed exempt from institutional review board approval from the University of Connecticut Institutional Review Board.

### Data Analysis

We performed a conventional content analysis of posts from the BGR Facebook page using a data-driven inductive framework approach in which content was coded into categories representing the implied intent of the post [40]. As a first step, a pair of investigators reviewed the posts, identified emerging themes, and developed a thematic framework. Following this process, the study team developed a codebook with 10 themes that were then applied to the first 50 posts. The 50 posts were then independently coded by 2 investigators who made refinements to the codebook based on areas of discrepant coding. The remaining 347 posts were double coded by 2 investigators. Interrater reliability was 89.7% and Cohen κ coefficient was 0.91 [41].

We calculated descriptive statistics for reactions (likes, love, wow, and care), comments, shares, and total engagement for each category (see Table 1). To evaluate the statistical association between the number of engagements post received and post types, generalized linear models were generated. Specifically, to account for the nature of the outcome variable (counts of the number of engagements), we used negative binomial regression models with 1 main predictor, post type, and time of posting as a covariate. Likelihood-ratio chi-squared statistics (Table 2) were used to evaluate the significance of post type. Three types of reactions (haha, angry, and sad) had insufficient frequency to compare across post types. Quantitative analyses were completed in R (version 4.1.0; R Core Team).

**Table 1:** Engagement data on Facebook posts.

Facebook post code			Likes (n=33,560)		Love (n=10,968)		Wow (n=82)		Haha (n=307)		Sad (n=10)		Angry (n=5)		Care (n=321)		Comments (n=5019)		Shares (n=5082)		Total engagements (n=55,354)
**Announcements (ref)**																					
	Engagements, n	3577		1662		19		5		2		3		37		1659		998		7962	
	Engagements, mean (SD)	59.3 (81.7)		26.9 (47.0)		0.3 (1.0)		0.1 (0.3)		0.0 (0.2)		0.0 (0.2)		0.6 (1.3)		26.7 (59.4)		16.0 (16.7)		129.9 (170.7)	
**Goals** **or** **motivational**																					
	Engagements, n	6961		2186		3		274		2		0		79		700		1285		11,490	
	Engagements, mean (SD)	107.5 (134.8)		33.7 (34.0)		0.0 (0.2)		4.2 (19.6)		0.0 (0.2)		0.0 (0.0)		1.2 (3.9)		11.7 (16.4)		19.9 (24.1)		160.1 (125.2)	
**Shout-outs**																					
	Engagements, n	14,858		4579		40		2		4		1		121		1861		802		22,268	
	Engagements, mean (SD)	121.6 (73.7)		37.5 (30.6)		0.3 (0.7)		0.0 (0.2)		0.0 (0.2)		0.0 (0.0)		1.0 (1.5)		14.8 (22.9)		6.5 (9.6)		181.7 (116.7)	
**Holidays** **or** **greetings**																					
	Engagements, n	594		178		1		0		1		0		6		28		97		905	
	Engagements, mean (SD)	74.3 (36.3)		22.3 (11.4)		0.1 (0.4)		0.0 (0.0)		0.1 (0.4)		0.0 (0.0)		0.8 (0.7)		3.5 (2.1)		12.1 (10.4)		113.1 (50.7)	
**Sponsored** **or** **ads**																					
	Engagements, n	1905		679		1		0		0		0		8		125		545		3263	
	Engagements, mean (SD)	35.3 (27.2)		12.6 (12.2)		0.0 (0.1)		0.0 (0.0)		0.0 (0.0)		0.0 (0.0)		0.1 (0.4)		2.3 (3.3)		10.1 (10.2)		60.4 (48.6)	
**Health related**																					
	Engagements, n	3046		800		14		1		1		1		33		351		883		5130	
	Engagements, mean (SD)	64.8 (54.5)		17.0 (17.4)		0.3 (1.8)		0.0 (0.1)		0.0 (0.1)		0.0 (0.1)		0.7 (1.0)		7.5 (13.0)		18.8 (22.0)		109.1 (91.6)	
**Lived Black experience**																					
	Engagements, n	1447		472		2		2		0		0		12		103		238		2276	
	Engagements, mean (SD)	62.9 (39.7)		20.5 (15.9)		0.1 (0.3)		0.1 (0.3)		0.0 (0.0)		0.0 (0.0)		0.5 (0.9)		4.5 (5.2)		10.3 (9.3)		99.0 (61.9)	
**Self-care**																					
	Engagements, n	1172		412		2		23		0		0		25		192		234		2060	
	Engagements, mean (SD)	78.1 (46.6)		27.5 (25.4)		0.1 (0.4)		1.5 (4.1)		0.0 (0.0)		0.0 (0.0)		1.7 (2.4)		12.8 (23.8)		15.6 (16.4)		137.3 (77.5)	
χ_7_^2^			119.37		63.995		23.73		—^b^		—		—		35.06		80.55		71.28		80.99
*P* value			*<.001^a^*		*<.001^a^*		*<.001^a^*		—		—		—		*<.001^a^*		*<.001^a^*		*<.001^a^*		*<.001^a^*

^a^Italics indicate statistical significance (*P*<.05).

^b^Not available due to insufficient frequencies of interactions.

**Table 2:** Likelihood ratio chi-square statistics for post type from negative binomial generalized linear models predicting post engagement. All models also included time of post as a covariate.

Outcome or model	Likelihood-ratio, χ_7_^2^	*P* value
Total engagements	80.99	*<.001^a^*
Likes	119.37	*<.001^a^*
Love	63.995	*<.001^a^*
Wow	23.73	*<.001^a^*
Haha	—^b^	—
Sad	—	—
Angry	—	—
Care	35.06	*<.001^a^*
Comments	80.55	*<.001^a^*
Shares	71.28	*<.001^a^*

^a^Boldface indicates statistical significance (<.05).

^b^Not available due to insufficient frequencies of interactions.

## Results

A total of 397 unique Facebook posts were observed on the BGR Facebook page in the 6-month period between June 1, 2021, and December 31, 2021, which amounted to an average of 1.86 posts per day. The most common type of post (n=122, 31%) was “shout-outs,” which we defined as posts that celebrated achievements, milestones, and other special moments among members (Table 3). The next most common type of post was goal or motivational posts (n=65, 16%), which were defined as posts that discussed goals and intentions or included motivational quotes. The next most common type of post was announcements (n=63, 16%), which we defined as posts that announced events, registration deadlines for events or cancellations, logistics, and other information regarding events. The next most common type of post was sponsored or ad posts (n=54, 14%), which were posts promoting a business or product to the community. Many of the sponsored or ad posts were health- or running related (eg, running shoes or healthy food ads). Next were health-related posts (n=47, 12%), the examples of which include posts that share advice, education, or experiences with health-related behaviors (eg, exercise, sleep, weight, nutrition, hydration, injury prevention, stress, COVID-19, or mental health). The 3 least common types of posts were those pertaining to the lived Black experience (eg, racism, discrimination, social justice; n=23, 6%), self-care (eg, taking time for oneself or taking actions to improve one’s wellness; n=15, 4%) and holidays or greetings, (eg, greetings for special occasions or holidays; n=8, 2%).

**Table 3:** List of codes.

Code	Posts, n (%)	Definition
Shout-outs	122 (31)	Posts that mention an individual’s or a group’s achievements, milestones, birthdays, anniversaries, and so forth.
Goals or motivational	65 (16)	Posts that discussed goals, intentions, and motivational quotes encouraging achieving goals.
Announcements	63 (16)	Any post that is announcing events, registration, cancellations, logistics, or other urgent information. The purpose is to give the community information about upcoming running events and inviting members to register.
Sponsored or ads	54 (14)	Promoting a business or product to the community. The purpose of the post is to feature a business or product, to get people to buy something, or patronize a business.
Health related	47 (11)	Posts that share advice, education, or experiences with health related behaviors including but not limited to exercise, sleep, nutrition, hydration, injury prevention, stress reduction, COVID-19, or a health condition (eg, asthma and diabetes) or a mental health condition (eg, depression).
Lived Black experience	23 (6)	Posts discussing racism, police brutality against African Americans, dangers of running while Black, Black health, haircare, and Black history.
Self-care	15 (4)	Posts that discuss taking time for oneself or taking actions to improve one’s wellness (eg, spirituality)
Holidays or greetings	8 (2)	Holiday-related messages, just serve the purpose of passing along greetings for special occasions, holidays, and so forth.

A total of 55,354 engagements were observed across 397 posts. Approximately 82% (n=45,253) of engagements were reactions (eg, like, love, wow, haha, sad, angry, and care), 9.1% (n=5019) were replies or comments, and 9.2% (n=5082) were shares. We observed a statistically significant difference in total engagement between post types (χ_7_^2^=80.99, *P*<.001). The post type with the highest mean engagement per post was shout-outs (mean 181.7, SD 116.7), followed by goals or motivational posts (mean 160.1, SD 125.2), self-care posts (mean 137.3, SD 77.5), announcements (mean 129.9, SD 170.7), holidays or greetings posts (mean 113.1, SD 50.7), health related posts (mean 109.1, SD 91.6), the lived Black experience (mean 181.7, SD 116.7), and sponsored or ad posts (mean 60.4, SD 48.6). In terms of reactions, “like” (n=33,560, 74%) was by far the most common, followed by “love” (n=10,967, 24%), “care” (n=321, 0.7%), “haha” (n=307, 0.7%), “wow” (n=82, 0.2%), “sad” (n=10, 0.02%), and “angry” (n=5, 0.01%). Significant differences were found between groups for all reaction types except for the “haha,” “sad,” and “angry” because cells were too small to compare.

The most common posts that received comments were health related (n=1861, 3%), announcements (n=1659, 33%), and goals or motivational (n=700, 14%). The next most common types of posts to receive comments were shout-outs (n=351, 7%), the lived Black experience (n=192, 4%), and self-care (n=125, 3%). The least common posts to receive comments were sponsored or ads (n=103, 2%) and holidays or greetings (n=28, 0.6%). Goals or motivational posts (n=1285, 25%), announcements (n=998, 20%), and shout-outs (n=883, 17%) were the most frequently shared. The next common posts to be shared were health related (n=802, 16%), self-care (n=545, 11%), and sponsored or ads (n=238, 5%). The least common posts shared were the lived Black experience (n=234, 5%) and holidays or greetings (n=97, 2%). See Table 1 for engagement data on Facebook posts across all 8 codes.

## Discussion

### Principal Results

Our content analysis of 397 unique posts during a 6-month period in the BGR public Facebook page revealed that the most popular type of posts acknowledged members’ achievements, motivated members to get active, and announced active events. Posts also included the content on health information across a variety of topics, self-care, and Black culture and experiences. Overall, results suggest that the BGR Facebook page provides PA resources and social support, which are known predictors of PA in Black women [9]. Future research should examine member characteristics of PA communities on Facebook and the impact of following and participating in these communities on PA behavior. Although public Facebook communities about PA likely attract many users who are already physically active, given the public nature, users’ friends can see their friends’ group membership and engagement in the community, which might motivate them to join as well. Studies should also explore how one’s engagement in public Facebook pages about health influence their friends’ interest in those pages and their engagement in PA.

Shout-outs, which are posts that highlighted member achievements and promoted inclusivity and community, were the most popular type of posts, which are encouraging given that Black women have been historically excluded from wellness communities, such as yoga [42,43]. Engagement data revealed that positive sentiment reactions represented the vast majority (n=45,238, 82%) of reactions (eg, likes, love, haha, and care), which suggest that the content being produced largely generates positive feelings. That said, negative sentiment reactions can also provide support. For example, sad or angry reactions to a post describing one’s experience with racism also convey support. Future studies should explore the content of group member comments to better understand the types of conversations that are generated as well as the sentiment of those conversations. Our findings suggest that the BGR Facebook page promotes a positive space that features women of varying sizes, skin tones, and physical abilities. Social media platforms provide an opportunity to create spaces that are safe for Black women to engage around health topics.

BGR Facebook posts appeared to demonstrate cultural awareness regarding issues that are important to Black women. For example, some posts alluded to the management of hair, which previous studies have documented as a barrier to PA in Black women including how the money and time required to restyle and maintain hair following a workout [44-47]. Hair in the Black community is a complicated phenomenon that, on the one hand, is a critical aspect of identity, self-esteem, and worth, but on the other hand, has been associated with discrimination [48] and microaggressions to an extent that many states have had to pass legislation to prohibit hair discrimination [49-51]. Future research should examine the impact of such role modeling of hair care during and after exercise and promotion of social norms around acceptable hairstyles in web-based exercise communities, as these might have a substantial effect on reducing PA barriers in Black women.

Almost 6% of posts addressed some aspect of the Black experience including topics such as racism and discrimination. For example, some posts addressed the 2020 murder of Ahmaud Arbery, a Black runner in Georgia who was killed by White vigilantes. The effect of this murder on the PA habits in the Black community is unknown, but robust literature reveals that lack of perceived safety is a substantial barrier to PA in Black communities [11]. This can be attributable to perceptions regarding the lack of neighborhood safety [52,53] as well as fears regarding racial discrimination and safety while running in White neighborhoods [54], and these fears were likely intensified by the murder of Ahmaud Arbery. Studies should examine how engagement around these topics in web-based communities like BGR may buffer the effects of such events on exercise motivation in Black women. To the extent that these communities provide an outlet to discuss concerns, safety measures, and connect users who would like to exercise together, they may facilitate greater comfort about exercising in public as well as ideas for places where Black women can exercise safely.

Other examples of Black experience that appeared in posts and elicited engagement was the social activism content that appeared in announcement posts. For example, 1 BGR post encouraged runners to join the eRace Racism Virtual Challenge, which is a run, walk, or hike event to raise money to combat racism. Other posts announced events and discussed walks or runs for civil rights and racial injustice. Future research should examine how to attach web-based social activism [55], an important community value, to PA in Black women as a way to enhance PA motivation while also engaging in activism for causes that directly affect their community.

Our content analysis also revealed that the BGR community is a source of health education on a variety of topics besides PA (eg, weight loss, the dangers of ultraviolet sun exposure, and mental health). More than 10% of the posts were of this nature. Facebook communities such as BGR may present opportunities to disseminate evidence-based health education and promotion programming—particularly given the strong infrastructure, the interest by group members, the group cohesion that is generated within social networks, and the mission of BGR to improve health. Notably, weight loss was among the most common health-related post topics. Because Black women have a disproportionately high prevalence of obesity (56.9%) [56], communities such as this may provide opportunities for partnership with experts in behavioral weight management to provide culturally relevant programming. Future studies should explore the interest of members of PA-focused web-based communities for Black women in more formal programming for weight management.

Previous studies have identified interpersonal barriers to PA, including poor social support or PA partners [11]. The web-based community of BGR addresses these barriers by offering a community of fellow Black women who are engaging in PA and supporting each other in their efforts to be active. The local chapters within the BGR community provide an additional opportunity for social support and community by offering in-person running and walking opportunities to members. BGR and other web-based communities are also uniquely positioned to provide knowledge and education about running safety and health education, which have also been cited as barriers to engaging in PA among Black women [11]. It is also possible that web-based communities such as BGR may provide motivation to exercise or engage in PA, which is an important aspect to PA engagement [57]. While there is extensive literature on barriers to PA among Black women (eg, motivation, cost, childcare, social support, safety, among others) [11,57,58], there is limited research on how people are engaging with these communities and how and if social media communities can ameliorate these barriers. Moreover, there is a critical research gap on the potential mechanisms and applicable theoretical frameworks regarding how social media engagement relates may relate to behavior change in the context of web-based communities such as BGR. For example, might engagement in social media communities such as BGR increase self-efficacy [59], thereby increasing the likelihood of PA engagement? Could principles of the transtheoretical model of change [60] explain how participants may become move from contemplative to ready to engage in PA? Might engagement with social media communities influence attitudes to engaging in PA, as seen in other health behaviors [61]? There is a need for further exploration in regard if and how social media engagement is related to engagement or lack of engagement in PA.

### Limitations

Some limitations should be considered when interpreting the results of this study. It is possible that members engaged in the BGR community are likely to be more physically active or already interested in PA than in the general population. Second, we cannot confirm that this group is representative of all Black women; thus, engagement data may not be generalizable to all Black women. In addition, engagement data may be an underestimate given that they reflect the engagement that occurred up until the day we extracted it from the page. However, because the vast majority of engagement (eg, likes, comments, and shares) on Facebook posts occur within the first 48 hours of the posting, we likely captured the majority of probable engagement [62].

### Conclusions

The findings offer a critical addition to the dearth of literature on web-based communities serving Black women, social media, and PA. BGR serves as a space for social connection, health education, and social support and engagement around PA. Future research should explore more about the potential links between engagement in social media posts and engaging in PA and also should examine how health promotion researchers can partner with web-based communities around health topics and reduce disparities in PA engagement, particularly among Black women.

## Abbreviations

BGR: Black Girls Run
PA: physical activity

## Data Availability

The data sets generated and analyzed during this study are available from the corresponding author on reasonable request.
